# Blood lead and cadmium levels are negatively associated with bone mineral density in young female adults

**DOI:** 10.1186/s13690-021-00636-x

**Published:** 2021-06-25

**Authors:** Jianfeng Lu, Ji Lan, Xiao’e Li, Zhongxin Zhu

**Affiliations:** 1 Department of Medical Administration, The First People’s Hospital of Xiaoshan District, Xiaoshan First Affiliated Hospital of Wenzhou Medical University, Hangzhou, 311200 Zhejiang China; 2 Department of Emergency, The First People’s Hospital of Xiaoshan District, Xiaoshan First Affiliated Hospital of Wenzhou Medical University, Hangzhou, 311200 Zhejiang China; 3 Department of Hematology, The First People’s Hospital of Xiaoshan District, Xiaoshan First Affiliated Hospital of Wenzhou Medical University, Hangzhou, 311200 Zhejiang China; 4 Department of Osteoporosis Care and Control, The First People’s Hospital of Xiaoshan District, Xiaoshan First Affiliated Hospital of Wenzhou Medical University, Hangzhou, 311200 Zhejiang China

**Keywords:** Lead, Cadmium, Bone health, NHANES

## Abstract

**Background:**

The organ toxicities of lead and cadmium have been extensively studied; however, studies of their toxic effects on bone remain limited, especially in young adults. The objective of this study was to examine the associations of blood lead levels (BLL) and blood cadmium levels (BCL) with bone mineral density (BMD) among young adults.

**Methods:**

We performed a cross-sectional study using the National Health and Nutrition Examination Survey 2011–2018 database. Because of the skewed distribution, BLL and BCL were Ln-transformed for analysis. Weighted multivariate regressions were performed to evaluate the associations between LnBLL and LnBCL and lumbar BMD. Subgroup analyses were further performed.

**Results:**

A total of 3234 participants aged 20–35 years were included in this study. No significant association between LnBLL and lumbar BMD was found (β = − 5.6, 95%CI: − 13.5–2.3). However, in the subgroup analysis stratified by sex, this association became negative in women (β = − 18.2, 95%CI: − 29.9– − 6.4). Moreover, this negative association was more prominent in female blacks (β = − 35.5, 95%CI: − 63.4– − 7.6). On the other hand, a negative association between LnBCL and lumbar BMD was found (β = − 7.4, 95%CI: − 14.0– − 0.8). In the subgroup analysis stratified by sex, this negative association only existed in women (β = − 18.7, 95%CI: − 28.0– − 9.5). Moreover, this negative association was more prominent in female whites (β = − 31.1, 95%CI: − 46.2– − 16.1).

**Conclusions:**

Our finding showed that both BLL and BCL were independently and negatively associated with lumbar BMD among young females, but not among young males.

## Background

Osteoporosis is a chronic systemic skeletal disorder characterized by decreased bone mineral density (BMD) and impaired microarchitecture, ultimately predisposing individuals to fragility fractures [1, 2]. Some metals including copper, iron, and zinc are necessary to maintain normal physiological functions of bones, but heavy metals such as lead (Pb) and cadmium (Cd) may be associated with osteoporosis and related fractures [3, 4].

As typical heavy metals, Pb and Cd are both of great concern due to their various deleterious health impacts. These two toxic metals have been included in the top 10 chemicals of major public health concern by the World Health Organization (WHO) [5]. Although the organ toxicities of Pb and Cd have been extensively studied, their toxic effects on bone remain limited, especially in young adults. We hypothesized that higher exposure of Pb or Cd is associated with lower BMD in young adults. Therefore, we investigated the associations of blood lead levels (BLL) and blood cadmium levels (BCL) with BMD in a representative sample of US adults aged from 20 to 35 years using the National Health and Nutrition Examination Survey (NHANES) 2011–2018 database.

## Materials and methods

### Study population

Data from the recent 8 years (2011–2018) of NHANES were analyzed in the present study. The participants of NHANES were from a representative of the non-institutionalized civilian population of US, with a complex multistage and stratified sampling design. The details of this program have been described previously [6].

A total of 6070 young adults aged from 20 to 35 years were enrolled from the NHANES 2011–2018 database. After exclusion of 2047 participants without BLL or BCL data, 763 participants without lumbar BMD data, and 26 participants with rheumatoid arthritis, 3234 subjects remained in the final analysis.

The research ethics review board of the National Center for Health Statistics (NCHS) approved all NHANES study protocols, and written informed consents were obtained from all participants.

### Study variables

The exposure variables were BLL and BCL. Metal assays for both BLL and BCL were conducted with whole blood specimens at the Division of Laboratory Sciences within the National Center for Environmental Health, using inductively coupled plasma mass spectrometry (ICP-MS; CDC method No. ITB0001A) [7].

The outcome variable was lumbar BMD. The measurement of lumbar BMD was provided by dual-energy X-ray absorptiometry scans, administered by trained and certified radiology technologists, using Hologic Discovery model A densitometers (Hologic, Inc., Bedford, Massachusetts).

Multivariate models contain variables that might confound the links between BLL, BCL and BMD. The data on age, sex, race, education level, income to poverty ratio, smoking behavior, and moderate recreational activities were obtained from questionnaires. Recreational activities was based on the question “do any moderate-intensity sports, fitness, or recreational activities that cause a small increase in breathing or heart rate such as brisk walking, bicycling, swimming, or golf for at least 10 minutes continuously?” Body mass index (BMI) was calculated by weight/height^2^, which were measured in the mobile examination center. The data on serum albumin, blood urea nitrogen, serum uric acid, serum phosphorus, and serum calcium were obtained from standard biochemistry profile analysis with a Beckman Synchron LX20. The detailed information on these covariates are publicly available on the NHANES website (www.cdc.gov/nchs/nhanes/).

### Statistical analyses

All statistical analyses were conducted using EmpowerStats software (X&Y Solutions, Boston, MA) and statistical software R (version 3.4.3), with weights calculated as recommended by analytic guidelines edited by NCHS [8]. The BLL and BCL were Ln-transformed for analysis because the distributions of values for BLL and BCL were highly skewed. Multivariate regressions were performed to evaluate the associations between BBL, BCL and lumbar BMD. Three models were built: unadjusted model, minimally adjusted model (adjusted for age, sex, and race), and fully adjusted model (adjusted for all the covariates listed in Table 1). Subgroup analyses stratified by sex and race were further performed. A *p*-value < 0.05 was considered statistically significant.

**Table 1:** The characteristics of participants

	Men (*n* = 1737)	Women (*n* = 1497)	*P* value
Age (years)	27.4 ± 4.5	27.3 ± 4.6	0.401
Race/Ethnicity (%)			0.676
Non-Hispanic White	58.6	58.2	
Non-Hispanic Black	11.7	12.5	
Mexican American	11.6	10.5	
Other race/ethnicity	18.0	18.8	
Education level (%)			< 0.001
Less than high school	11.7	8.3	
High school	25.8	20.0	
More than high school	62.5	71.7	
Income to poverty ratio	2.7 ± 1.6	2.5 ± 1.6	0.003
Moderate recreational activities (%)			< 0.001
Yes	47.4	53.4	
No	52.6	46.6	
Smoked > = 100 cigarettes in life (%)			< 0.001
Yes	43.0	28.6	
No	57.0	71.4	
Body mass index (kg/m^2^)	27.9 ± 6.2	28.3 ± 7.7	0.043
Serum albumin (g/L)	45.1 ± 3.0	42.2 ± 3.2	< 0.001
Blood urea nitrogen (mmol/L)	4.7 ± 1.4	3.9 ± 1.2	< 0.001
Serum uric acid (μmol/L)	359.0 ± 71.9	272.0 ± 61.2	< 0.001
Serum phosphorus (mmol/L)	1.2 ± 0.2	1.2 ± 0.2	0.267
Serum calcium (mmol/L)	2.4 ± 0.1	2.3 ± 0.1	< 0.001
Blood lead (μmol/L)	0.05 ± 0.07	0.03 ± 0.03	< 0.001
Blood cadmium (μmol/L)	3.23 ± 4.12	3.56 ± 4.78	0.038
Lumbar bone mineral density (mg/cm^2^)	1041.0 ± 145.5	1056.7 ± 127.3	0.001

Mean ± SD for continuous variables: *P* value was calculated by weighted linear regression model

% for Categorical variables: *P* value was calculated by weighted chi-square test

## Results

A total of 3234 participants aged 20–35 years were included in this study. The weighted distributions of the characteristics according to sex are shown in Table 1. Compared with men, women had higher levels of education, higher moderate recreational activities, higher BMI, higher BCL, and higher BMD, but lower income to poverty ratio, lower percentage of smoked > = 100 cigarettes in life, and lower levels of serum albumin, blood urea nitrogen, serum uric acid, serum calcium, and lower BLL.

The associations between LnBLL and lumbar BMD by multivariate regression analyses are shown in Table 2. No significant associations were found in all three models. However, in our subgroup analysis stratified by sex, this association became negative in women in all three models (unadjusted model: β = − 12.4, 95% CI: − 23.7– − 1.1; minimally adjusted model: β = − 12.6, 95% CI: − 24.0– − 1.3; fully adjusted model: β = − 18.2, 95% CI: − 29.9– − 6.4). Moreover, this negative association was more prominent in female blacks (Table 3).

**Table 2:** Association between LnBLL and lumbar bone mineral density (mg/cm^2^)

	Unadjusted model β (95% CI)	Minimally adjusted model β (95% CI)	Fully adjusted model β (95% CI)
Per 1 μmol/L increase	−9.2 (−16.4, −2.0)^*^	−4.5 (− 12.1, 3.0)	−5.6 (− 13.5, 2.3)
Stratified by sex			
Men	−1.6 (− 12.1, 8.8)	1.2 (− 9.0, 11.4)	3.3 (− 7.5, 14.0)
LnBLL (Quartile)			
Q1	Reference	Reference	Reference
Q2	−23.1 (−42.3, −3.9)	− 21.6 (− 40.2, − 2.9)	−18.0 (− 37.0, 1.1)
Q3	−9.6 (− 28.9, 9.7)	− 6.2 (− 24.9, 12.6)	−5.8 (− 25.2, 13.6)
Q4	−8.8 (− 27.8, 10.2)	− 2.8 (− 21.5, 15.8)	2.7 (− 17.0, 22.4)
P for trend	0.635	0.850	0.534
Women	−12.4 (− 23.7, − 1.1)^**^	−12.6 (− 24.0, − 1.3)^**^	−18.2 (− 29.9, − 6.4)^***^
LnBLL (Quartile)			
Q1	Reference	Reference	Reference
Q2	15.3 (− 2.4, 33.0)	14.5 (− 3.0, 32.0)	13.7 (− 3.7, 31.1)
Q3	−2.6 (− 20.4, 15.2)	−3.3 (− 21.0, 14.3)	−9.0 (− 26.9, 8.9)
Q4	−6.8 (− 25.4, 11.9)	−7.0 (− 25.8, 11.7)	− 17.3 (− 36.6, 2.0)
P for trend	0.441	0.415	0.099

Unadjusted model: no covariates were adjusted

Minimally adjusted model: age, sex, and race were adjusted

Fully adjusted model: age, sex, race, education level, income to poverty ratio, smoking behavior, body mass index, moderate recreational activities, serum albumin, blood urea nitrogen, serum uric acid, serum phosphorus, and serum calcium were adjusted

*Abbreviation*: *BLL* Blood lead levels

^*^ *P* < 0.05, ^**^ *P* < 0.01, ^***^ *P* < 0.001

**Table 3:** Association between LnBLL and lumbar bone mineral density (mg/cm^2^), stratified by sex and race

	Unadjusted model β (95% CI)	Minimally adjusted model β (95% CI)	Fully adjusted model β (95% CI)
Men			
Non-Hispanic White	5.6 (− 12.2, 23.4)	6.3 (− 11.6, 24.1)	8.1 (− 11.5, 27.6)
Non-Hispanic Black	−5.1 (− 29.4, 19.3)	−7.0 (− 32.0, 18.1)	−3.2 (− 30.3, 23.9)
Mexican American	−8.0 (− 29.2, 13.2)	− 9.4 (− 30.8, 12.1)	1.3 (− 21.7, 24.3)
Other race/ethnicity	−1.9 (− 19.2, 15.4)	−0.8 (− 18.4, 16.7)	7.5 (− 11.9, 27.0)
Women			
Non-Hispanic White	−11.2 (− 31.4, 9.0)	− 11.6 (− 31.8, 8.7)	−16.3 (− 37.7, 5.0)
Non-Hispanic Black	− 32.2 (− 58.5, − 6.0)^*^	−31.3 (− 58.2, − 4.4)^*^	− 35.5 (− 63.4, − 7.6)^*^
Mexican American	−27.1 (− 52.2, − 2.0)^*^	− 31.4 (− 56.8, − 5.9)^*^	−24.7 (− 51.9, 2.4)
Other race/ethnicity	2.1 (− 16.7, 20.9)	2.8 (− 16.0, 21.7)	−2.7 (− 22.2, 16.8)

Unadjusted model: no covariates were adjusted

Minimally adjusted model: age were adjusted

Fully adjusted model: age, education level, income to poverty ratio, smoking behavior, body mass index, moderate recreational activities, serum albumin, blood urea nitrogen, serum uric acid, serum phosphorus, and serum calcium were adjusted

*Abbreviation*: *BLL* Blood lead levels

^*^ *P* < 0.05, ^**^ *P* < 0.01, ^***^ *P* < 0.001

On the other hand, there was a negative association between LnBCL and lumbar BMD in the fully adjusted model (β = − 7.4, 95% CI: − 14.0– − 0.8), as shown in Table 4. In the subgroup analysis stratified by sex, this negative association only existed in women in the fully adjusted model (β = − 18.7, 95% CI: − 28.0– − 9.5), with a significant P for trend (*P* = 0.009). Moreover, this negative association was more prominent in female whites (Table 5).

**Table 4:** Association between LnBCL and lumbar bone mineral density (mg/cm^2^)

	Unadjusted model β (95% CI)	Minimally adjusted model β (95% CI)	Fully adjusted model β (95% CI)
Per 1 μmol/L increase	−2.3 (−7.8, 3.3)	−6.1 (− 11.6, − 0.6)^*^	−7.4 (− 14.0, − 0.8)^*^
Stratified by sex			
Men	2.2 (− 5.6, 10.0)	− 2.3 (− 9.9, 5.3)	1.7 (− 7.6, 11.0)
LnBCL (Quartile)			
Q1	Reference	Reference	Reference
Q2	− 6.3 (− 25.4, 12.7)	−11.4 (− 29.9, 7.1)	− 7.6 (− 26.3, 11.1)
Q3	−22.0 (− 40.3, − 3.6)	−27.0 (− 45.0, − 9.0)	−22.6 (− 41.8, − 3.4)
Q4	7.3 (− 12.1, 26.7)	−4.3 (− 23.2, 14.7)	3.3 (− 19.7, 26.3)
P for trend	0.927	0.234	0.550
Women	−10.4 (− 18.3, − 2.6)^**^	−12.2 (− 20.0, − 4.3)^**^	−18.7 (− 28.0, − 9.5)^***^
LnBCL (Quartile)			
Q1	Reference	Reference	Reference
Q2	−4.1 (− 22.1, 13.9)	−3.1 (− 21.0, 14.7)	− 5.8 (− 23.6, 12.1)
Q3	4.6 (− 12.8, 22.0)	6.9 (− 10.5, 24.3)	0.9 (− 16.8, 18.6)
Q4	− 20.6 (− 38.9, − 2.3)	− 23.6 (− 41.9, − 5.3)	− 36.5 (− 57.3, − 15.7)
P for trend	0.101	0.062	0.009

Unadjusted model: no covariates were adjusted

Minimally adjusted model: age, sex, and race were adjusted

Fully adjusted model: age, sex, race, education level, income to poverty ratio, smoking behavior, body mass index, moderate recreational activities, serum albumin, blood urea nitrogen, serum uric acid, serum phosphorus, and serum calcium were adjusted

*Abbreviation*: *BCL* Blood cadmium levels

^*^ *P* < 0.05, ^**^ *P* < 0.01, ^***^ *P* < 0.001

**Table 5:** Association between LnBCL and lumbar bone mineral density (mg/cm^2^), stratified by sex and race

	Unadjusted model β (95% CI)	Minimally adjusted model β (95% CI)	Fully adjusted model β (95% CI)
Men			
Non-Hispanic White	−6.5 (− 18.4, 5.5)	−6.2 (− 18.2, 5.9)	−4.2 (− 19.6, 11.1)
Non-Hispanic Black	7.1 (−11.5, 25.7)	6.7 (−12.0, 25.4)	20.3 (− 5.4, 46.1)
Mexican American	5.6 (−16.2, 27.5)	4.7 (− 17.2, 26.6)	− 3.1 (− 28.3, 22.1)
Other race/ethnicity	4.3 (− 10.8, 19.4)	4.8 (− 10.3, 20.0)	13.0 (− 5.2, 31.2)
Women			
Non-Hispanic White	−23.5 (−35.8, − 11.2)^***^	−24.1 (− 36.5, − 11.7)^***^	−31.1 (− 46.2, − 16.1)^***^
Non-Hispanic Black	2.1 (− 14.8, 18.9)	3.7 (− 13.6, 20.9)	−2.3 (− 24.2, 19.6)
Mexican American	11.8 (−15.2, 38.7)	8.3 (−19.3, 35.9)	−1.4 (− 30.6, 27.7)
Other race/ethnicity	12.4 (− 3.1, 27.9)	12.5 (− 3.0, 28.0)	5.6 (− 12.1, 23.2)

Unadjusted model: no covariates were adjusted

Minimally adjusted model: age were adjusted

Fully adjusted model: age, education level, income to poverty ratio, smoking behavior, body mass index, moderate recreational activities, serum albumin, blood urea nitrogen, serum uric acid, serum phosphorus, and serum calcium were adjusted

*Abbreviation*: *BCL* Blood cadmium levels

^*^ *P* < 0.05, ^**^ *P* < 0.01, ^***^ *P* < 0.001

**Fig. 1:**
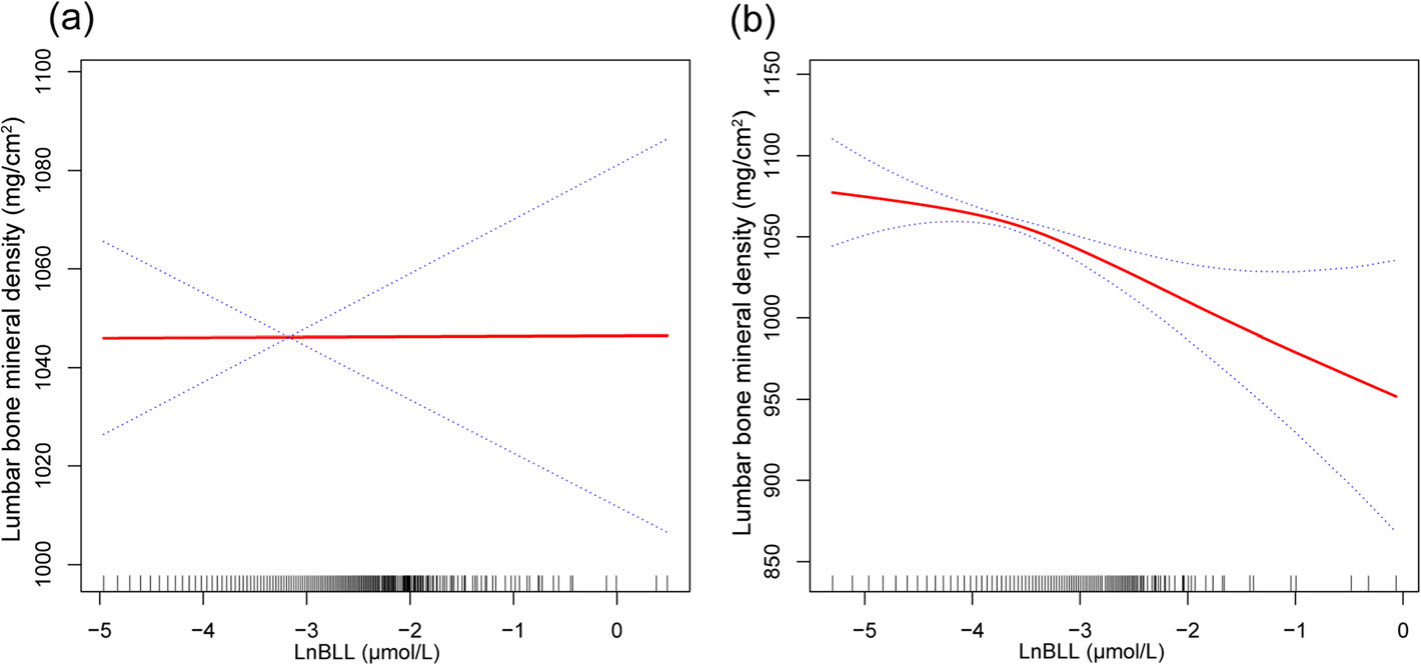
The associations between LnBBL and lumbar bone mineral density. **a** men. **b** women. Adjusted for age, race, education level, income to poverty ratio, smoking behavior, body mass index, moderate recreational activities, serum albumin, blood urea nitrogen, serum uric acid, serum phosphorus, and serum calcium. Abbreviations: BLL, blood lead levels

**Fig. 2:**
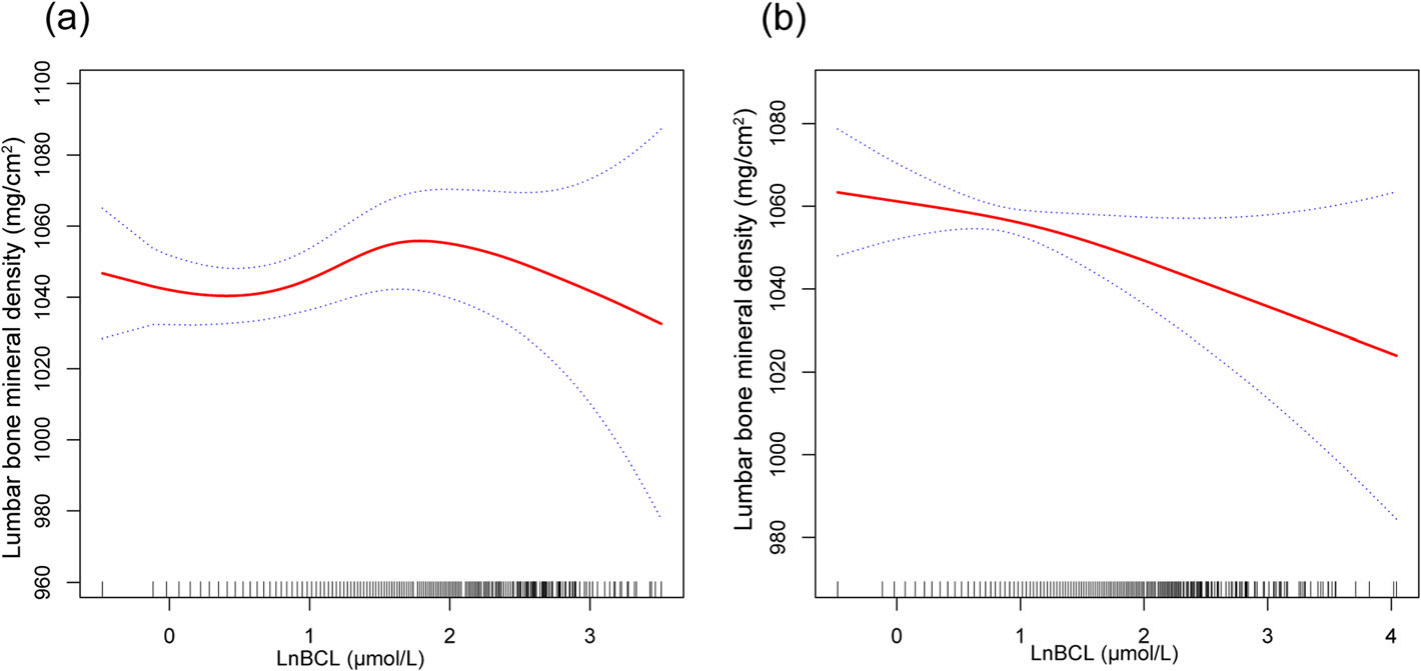
The associations between LnBCL and lumbar bone mineral density. **a** men. **b** women. Adjusted for age, race, education level, income to poverty ratio, smoking behavior, body mass index, moderate recreational activities, serum albumin, blood urea nitrogen, serum uric acid, serum phosphorus, and serum calcium. Abbreviations: BCL, blood cadmium levels

The associations between LnBLL, LnBCL and lumbar BMD were further confirmed by generalized additive models and smooth curve fittings (Figs. 1 and 2).

## Discussion

This study examined the association of BLL and BCL with lumbar BMD using a representative sample of US young adults enrolled from NHANES 2011–2018. Our results showed that both BLL and BCL were independently and negatively associated with lumbar BMD among females, but not among males.

Pb is a toxic heavy metal, and its widespread use has resulted in environmental pollution. Several epidemiological studies have reported the relationship between BLL and bone health, but with inconsistent conclusions. In a previous NHANES study (2013–2014) of 1859 adults aged ≥40 years, Wang et al. [9] found that lead exposure was associated with decreased BMD in premenopausal women. The results of another NHANES study (NHANES III) of adults aged ≥50 years revealed that BLL was inversely associated with BMD, but only among white participants [10]. A significant inverse association was also observed in another study from the Korea National Health and Nutrition Examination Survey 2008–2011 [11]. However, other studies have reported either no association [12], or a positive association [13].

On the other hand, exposure to Cd is broadly toxic and can cause negative impacts on human health [14]. The effects of Cd exposure on bone health remain controversial. In a population-based study from China, Chen et al. [15] found that cumulative Cd intake, estimated by a food survey, was significantly associated with decreased BMD in women, but no statistical significance was found in men. However, the results of the Swedish cohort of the Osteoporotic Fractures in Men (MrOS) study revealed that Cd in urine was negatively associated with BMD in elderly men [16]. These inconsistent findings on the associations of BLL and BCL with BMD may be attributed to the heterogeneity between these studies, including participant selection, BMD examination sites, Cd exposure measurement methods, study design, and control of confounding factors. The results of our subgroup analyses suggested that BLL and BCL were negatively associated with lumbar BMD in young females, but not in young males. Previous studies also have shown that Cd-induced bone damage is gender dependent [17, 18]. Current research on the associations between BLL, BCL and BMD in young adults is limited and future large-sample prospective studies are needed to confirm our results. Evidence from the MrOS study showed that the smoking-induced decrease in BMD is largely due to the mediating effect of cadmium [19]. This indicates that reducing the intake of smoking or secondhand smoke may be beneficial to the bone health of young adults.

The exact mechanism of Pb and Cd exposures on bone health remains unclear. Pb and Cd are two encountered heavy metals, that may exert direct effects on bone cells. A recent study in vitro showed that both Pb and Cd exposures impair human osteoblast cellular bioenergetics and generate redox stress, and decrease the secretory output from osteoblasts [20]. In this process, Cd was more cytotoxic [20]. In addition, compared to male animals, females were more susceptible to Cd in bone [21, 22]. One possible explanation is that Cd exposure can reduce the level of estrogen 2, which inhibits the osteoclast activity and reduce bone absorption [23]. Studies regarding the effects of Cd on bone microstructure also showed that Cd exposure was associated with reduced trabecular bone volume fraction, cortical thickness, and cortical area [24, 25]. The pathological mechanisms of bone loss induced by Pb and Cd require further research.

Our study combined four waves of the latest NHANES data. To the best of our knowledge, this study was the largest sample report to investigate the associations between BLL, BCL and BMD in young adults, with the NHANES’s rigorous quality control of the procedures. Additionally, we were able to perform valid subgroup analysis because of the large sample size. However, there are some limitations which should be noted. First, causal relationships of BLL and BCL with BMD could not be determined due to the cross-sectional design of this study. Second, several covariant data were obtained through self-reports, which might be susceptible to self-report bias. However, the data were collected by well-trained interviewers with standardized protocols. Third, NHANES 2011–2012 and 2015–2016 surveys lacked data on femoral neck and total hip BMD. Therefore, we could not assess the relationships between BLL, BCL and BMD of these skeletal sites in this study. Fourth, in NHANES surveys, data regarding prednisone or cortisone taken and family history of osteoporosis (parents ever told they had osteoporosis) were collected only for participants aged 40 years and over. Therefore, we could not take these confounding factors into consideration in our analysis. Last, NHANES 2011–2018 surveys lack the data of bone turnover markers, which would be highly pertinent and interesting to assess in conjunction with the BMD data. This should be considered for future studies.

## Conclusions

Both BLL and BCL were independently and negatively associated with lumbar BMD among young females, but not among young males. Further studies are warranted to elucidate the potential mechanisms underlying the relationships between Pb, Cd and bone health.

## Data Availability

The data of this study are publicly available on the NHANES website.

## Abbreviations

BLL: Blood lead levels
BCL: Blood cadmium levels
BMD: Bone mineral density
Pb: Lead
Cd: Cadmium
WHO: The World Health Organization
NHANES: The National Health and Nutrition Examination Survey
NCHS: The National Center for Health Statistics
BMI: Body mass index
Q: Quartiles
